# (*E*)-4-Meth­oxy-*N*′-[(6-methyl-4-oxo-4*H*-chromen-3-yl)methyl­idene]benzo­hydrazide monohydrate

**DOI:** 10.1107/S1600536814013713

**Published:** 2014-06-18

**Authors:** Yoshinobu Ishikawa, Kohzoh Watanabe

**Affiliations:** a School of Pharmaceutical Sciences, University of Shizuoka, 52-1 Yada, Suruga-ku, Shizuoka 422-8526, Japan

## Abstract

In the title hydrate, C_19_H_16_N_2_O_4_·H_2_O, the 4*H*-chromen-4-one segment is slightly twisted, with a dihedral angle between the two six-membered rings of 3.30 (5)°. The dihedral angles between the plane of the pyran­one ring and the hydrazide plane and between the planes of the pyran­one ring and the benzene ring of the *p*-meth­oxy­benzene unit are 26.69 (4) and 2.23 (3)°, respectively. The mol­ecule is connected to the solvent water mol­ecule by an N—H⋯O hydrogen bond. In the crystal, there are π–π stacking inter­actions between centrosymmetrically related pyran­one rings [centroid–centroid distance = 3.5394 (9) Å], as well as bridges formed by the water mol­ecules *via* O—H⋯O hydrogen bonds.

## Related literature   

For the biological activity of related compounds, see: Khan *et al.* (2009 ▶); Tu *et al.* (2013 ▶). For related structures, see: Ishikawa & Watanabe (2014*a* ▶,*b* ▶). 
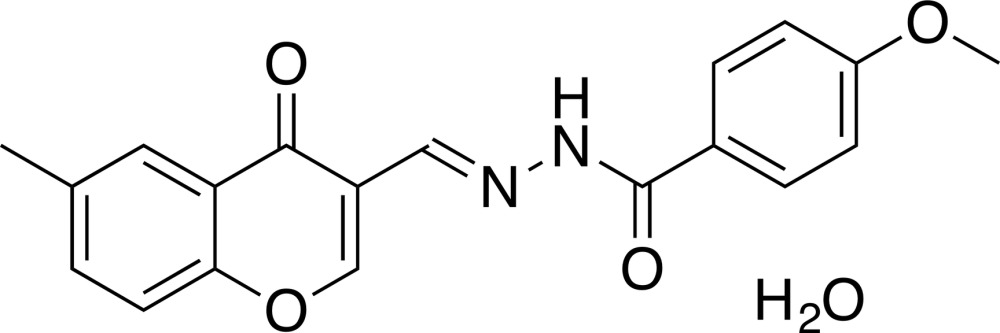


## Experimental   

### 

#### Crystal data  

C_19_H_16_N_2_O_4_·H_2_O
*M* *_r_* = 354.36
Triclinic, 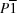

*a* = 7.6228 (13) Å
*b* = 10.809 (3) Å
*c* = 11.260 (2) Å
α = 116.339 (14)°
β = 94.258 (14)°
γ = 96.190 (16)°
*V* = 818.8 (3) Å^3^
*Z* = 2
Mo *K*α radiation
μ = 0.11 mm^−1^
*T* = 100 K
0.48 × 0.35 × 0.13 mm

#### Data collection  

Rigaku AFC-7R diffractometer
4581 measured reflections
3748 independent reflections
3219 reflections with *F* ^2^ > 2σ(*F* ^2^)
*R*_int_ = 0.008
3 standard reflections every 150 reflections intensity decay: −0.9%

#### Refinement  

*R*[*F* ^2^ > 2σ(*F* ^2^)] = 0.036
*wR*(*F* ^2^) = 0.104
*S* = 1.04
3748 reflections
245 parameters
H-atom parameters constrained
Δρ_max_ = 0.28 e Å^−3^
Δρ_min_ = −0.27 e Å^−3^

### 

Data collection: *WinAFC Diffractometer Control Software* (Rigaku, 1999 ▶); cell refinement: *WinAFC Diffractometer Control Software*; data reduction: *WinAFC Diffractometer Control Software*; program(s) used to solve structure: *SIR2008* (Burla *et al.*, 2007 ▶); program(s) used to refine structure: *SHELXL97* (Sheldrick, 2008 ▶); molecular graphics: *CrystalStructure* (Rigaku, 2010 ▶); software used to prepare material for publication: *CrystalStructure*.

## Supplementary Material

Crystal structure: contains datablock(s) General, I. DOI: 10.1107/S1600536814013713/mw2124sup1.cif

Structure factors: contains datablock(s) I. DOI: 10.1107/S1600536814013713/mw2124Isup2.hkl

Supporting information file. DOI: 10.1107/S1600536814013713/mw2124Isup3.cml

Additional supporting information:  crystallographic information; 3D view; checkCIF report

## Figures and Tables

**Table 1:** Hydrogen-bond geometry (Å, °)

*D*—H⋯*A*	*D*—H	H⋯*A*	*D*⋯*A*	*D*—H⋯*A*
O5—H17⋯O3^i^	0.86	1.99	2.8465 (13)	178
O5—H18⋯O2^ii^	0.87	1.96	2.8274 (16)	176
N2—H6⋯O5	0.88	2.07	2.9341 (14)	166

Symmetry codes: (i) 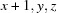; (ii) 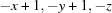.

**Fig. 1.**
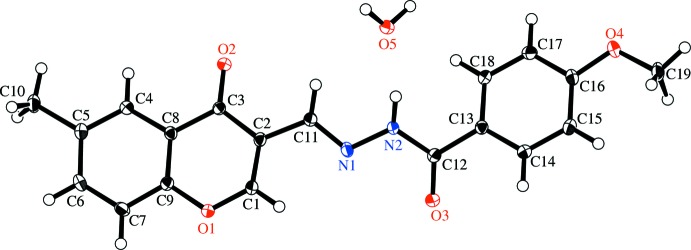
The molecular structure of the title compound, with displacement ellipsoids drawn at the 50% probability level. Hydrogen atoms are shown as small spheres of arbitrary radius.

**Fig. 2.**
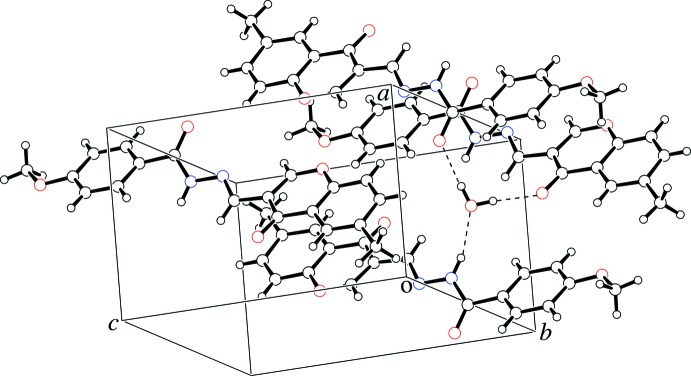
A crystal packing view of the title compound. Intermolecular N–H···O and O–H···O hydrogen bonds are represented by dashed lines.

## supplementary crystallographic information

### S1. Comment

Schiff base derivatives of 3-formyl chromones have attracted much attention due to their biological functions such as enzyme inhibition (Khan *et al.* 2009; Tu *et al.* 2013). We herein report the crystal structure of the title compound, which was obtained from the condensation reaction of 6-methyl-3-formylchromone with 4-methoxybenzoylhydrazide in benzene.

The 4*H*-chromen-4-one segment segment is slightly twisted with a dihedral angle between the two 6-membered rings of 3.30 (5)°. The dihedral angles between the pyranone ring and the hydrazide plane (C11/N1/N2/C12) and between the pyranone ring and the benzene ring of the *p*-methoxybenzene unit are 26.69 (4) and 2.23 (3)°, respectively. The molecule is connected to the solvent water molecule byan N–H···O hydrogen bond.

In the crystal, there are π-π stacking interactions between centrosymmetrically-related pyranone rings [centroid–centroid^i^ distance = 3.5394 (9) Å, i: –*x* + 1, –*y* + 1, –*z* + 1] as well as bridges formed by the water molecules *via* O–H···O hydrogen bonds (Fig. 2).

### S2. Experimental

4-Methoxybenzoylhydrazide (1.00 mmol), 6-methyl-3-formylchromone (1.00 mmol), and a few drops of acetic acid were dissolved in 25 mL of benzene and the mixture was refluxed with a Dean-Stark apparatus for 6 h. After cooling, the precipitates were collected, washed with *n*-hexane, and dried (yield 68.9%). ^1^H NMR (400 MHz, DMSO-*d*_6_): *δ* = 2.45 (s, 3H), 3.84 (s, 3H), 7.06 (d, 1H, *J* = 8.8 Hz), 7.63 (d, 1H, *J* = 8.3 Hz), 7.69 (dd, 1H, *J* = 2.5 and 8.3 Hz), 7.93 (d, 1H, *J* = 8.8 Hz), 7.94 (d, 1H, *J* = 2.5 Hz), 8.63 (s, 1H), 8.79 (s, 1H), 11.82 (s, 1H). DART-MS calcd for [C_19_H_16_N_2_O_4_ + H^+^]: 337.119, found 337.194. Single crystals suitable for X-ray diffraction were obtained by slow evaporation of an *N*,*N*-dimethylformamide solution of the title compound at room temperature.

### S3. Refinement

The C(*sp*^2^)- and N(*sp*^2^)-bound hydrogen atoms were placed in geometrical positions [C–H 0.95 Å, *U*_iso_(H) = 1.2*U*_eq_(C), N–H 0.88 Å, *U*_iso_(H) = 1.2*U*_eq_(N)], and refined using a riding model. Hydrogen atoms of methyl groups were found in a difference Fourier map, and a rotating group model was applied with distance constraint [C–H = 0.98 Å, *U*_iso_(H) = 1.2*U*_eq_(C)]. Hydrogen atoms of the water molecule were found in a difference Fourier map, and were refined using a riding model.

### Crystal data

**Table:**

C_19_H_16_N_2_O_4_·H_2_O	*Z* = 2
*M**_r_* = 354.36	*F*(000) = 372.00
Triclinic, *P*1	*D*_x_ = 1.437 Mg m^−^^3^
Hall symbol: -P 1	Mo *K*α radiation, λ = 0.71069 Å
*a* = 7.6228 (13) Å	Cell parameters from 25 reflections
*b* = 10.809 (3) Å	θ = 15.0–17.3°
*c* = 11.260 (2) Å	µ = 0.11 mm^−^^1^
α = 116.339 (14)°	*T* = 100 K
β = 94.258 (14)°	Block, colorless
γ = 96.190 (16)°	0.48 × 0.35 × 0.13 mm
*V* = 818.8 (3) Å^3^	

### Data collection

**Table:**

Rigaku AFC-7R diffractometer	θ_max_ = 27.5°
ω–2θ scans	*h* = −9→5
4581 measured reflections	*k* = −13→14
3748 independent reflections	*l* = −14→14
3219 reflections with *F*^2^ > 2σ(*F*^2^)	3 standard reflections every 150 reflections
*R*_int_ = 0.008	intensity decay: −0.9%

### Refinement

**Table:**

Refinement on *F*^2^	Secondary atom site location: difference Fourier map
*R*[*F*^2^ > 2σ(*F*^2^)] = 0.036	Hydrogen site location: inferred from neighbouring sites
*wR*(*F*^2^) = 0.104	H-atom parameters constrained
*S* = 1.04	*w* = 1/[σ^2^(*F*_o_^2^) + (0.0564*P*)^2^ + 0.2692*P*] where *P* = (*F*_o_^2^ + 2*F*_c_^2^)/3
3748 reflections	(Δ/σ)_max_ < 0.001
245 parameters	Δρ_max_ = 0.28 e Å^−^^3^
0 restraints	Δρ_min_ = −0.27 e Å^−^^3^
Primary atom site location: structure-invariant direct methods	

### Special details

**Table:**

Refinement. Refinement was performed using all reflections. The weighted *R*-factor (*wR*) and goodness of fit (*S*) are based on *F*^2^. *R*-factor (gt) are based on *F*. The threshold expression of *F*^2^ > 2.0 σ(*F*^2^) is used only for calculating *R*-factor (gt).

### Fractional atomic coordinates and isotropic or equivalent isotropic displacement parameters (Å^2^)

**Table:**

	*x*	*y*	*z*	*U*_iso_*/*U*_eq_	
O1	0.20207 (10)	0.53571 (9)	0.53711 (8)	0.01721 (18)	
O2	0.55749 (11)	0.68656 (9)	0.35944 (8)	0.01894 (19)	
O3	−0.21138 (10)	0.30160 (9)	−0.02159 (8)	0.02063 (19)	
O4	−0.10198 (11)	0.11287 (9)	−0.63143 (8)	0.02078 (19)	
O5	0.43347 (11)	0.31344 (10)	−0.10886 (9)	0.0217 (2)	
N1	0.11013 (12)	0.40533 (10)	0.13232 (9)	0.0151 (2)	
N2	0.09049 (12)	0.34737 (10)	−0.00533 (9)	0.0148 (2)	
C1	0.18957 (14)	0.48425 (12)	0.40352 (11)	0.0155 (3)	
C2	0.30132 (14)	0.52846 (11)	0.33784 (10)	0.0138 (3)	
C3	0.45241 (14)	0.63884 (11)	0.41270 (10)	0.0134 (2)	
C4	0.61596 (14)	0.78940 (11)	0.64525 (11)	0.0148 (3)	
C5	0.63334 (15)	0.83414 (11)	0.78214 (11)	0.0162 (3)	
C6	0.49795 (16)	0.78222 (12)	0.83435 (11)	0.0179 (3)	
C7	0.35334 (15)	0.68501 (12)	0.75278 (11)	0.0177 (3)	
C8	0.47079 (14)	0.68973 (11)	0.55867 (10)	0.0134 (3)	
C9	0.34401 (14)	0.63802 (11)	0.61494 (11)	0.0147 (3)	
C10	0.79455 (17)	0.93337 (13)	0.87393 (11)	0.0223 (3)	
C11	0.26801 (14)	0.46611 (11)	0.19139 (11)	0.0141 (2)	
C12	−0.07629 (14)	0.30143 (11)	−0.07521 (11)	0.0145 (3)	
C13	−0.08209 (14)	0.25000 (11)	−0.22219 (11)	0.0139 (2)	
C14	−0.21783 (15)	0.14353 (12)	−0.30858 (11)	0.0165 (3)	
C15	−0.22855 (15)	0.09151 (12)	−0.44696 (11)	0.0174 (3)	
C16	−0.10404 (15)	0.15125 (11)	−0.49879 (11)	0.0154 (3)	
C17	0.03025 (15)	0.26040 (12)	−0.41282 (11)	0.0159 (3)	
C18	0.04234 (14)	0.30807 (11)	−0.27641 (11)	0.0145 (3)	
C19	−0.23205 (17)	−0.00285 (13)	−0.72346 (11)	0.0229 (3)	
H1	0.0943	0.4108	0.3511	0.0186*	
H2	0.7036	0.8267	0.6091	0.0177*	
H3	0.5064	0.8150	0.9284	0.0214*	
H4	0.2628	0.6511	0.7894	0.0212*	
H5	0.3614	0.4707	0.1414	0.0169*	
H6	0.1845	0.3402	−0.0474	0.0177*	
H7	−0.3048	0.1056	−0.2725	0.0198*	
H8	−0.3192	0.0166	−0.5050	0.0208*	
H9	0.1135	0.3019	−0.4486	0.0191*	
H10	0.1356	0.3807	−0.2188	0.0174*	
H11A	0.8763	0.9571	0.8213	0.0268*	
H12B	0.8551	0.8888	0.9208	0.0268*	
H13C	0.7571	1.0187	0.9394	0.0268*	
H14A	−0.2229	−0.0838	−0.7069	0.0274*	
H15B	−0.2108	−0.0262	−0.8153	0.0274*	
H16C	−0.3515	0.0223	−0.7112	0.0274*	
H17	0.5408	0.3116	−0.0806	0.0412*	
H18	0.4419	0.3158	−0.1845	0.0441*	

### Atomic displacement parameters (Å^2^)

**Table:**

	*U*^11^	*U*^22^	*U*^33^	*U*^12^	*U*^13^	*U*^23^
O1	0.0149 (4)	0.0220 (4)	0.0133 (4)	−0.0023 (3)	0.0011 (3)	0.0079 (4)
O2	0.0155 (4)	0.0265 (5)	0.0147 (4)	−0.0021 (4)	0.0015 (3)	0.0105 (4)
O3	0.0131 (4)	0.0313 (5)	0.0159 (4)	0.0008 (4)	0.0022 (3)	0.0099 (4)
O4	0.0224 (5)	0.0252 (5)	0.0114 (4)	0.0014 (4)	0.0011 (3)	0.0060 (4)
O5	0.0146 (4)	0.0348 (5)	0.0165 (4)	0.0022 (4)	0.0025 (4)	0.0129 (4)
N1	0.0165 (5)	0.0164 (5)	0.0110 (5)	0.0030 (4)	0.0009 (4)	0.0050 (4)
N2	0.0125 (5)	0.0191 (5)	0.0101 (5)	0.0012 (4)	0.0011 (4)	0.0047 (4)
C1	0.0135 (5)	0.0181 (5)	0.0128 (5)	0.0012 (4)	0.0000 (4)	0.0058 (4)
C2	0.0123 (5)	0.0153 (5)	0.0126 (5)	0.0031 (4)	0.0002 (4)	0.0055 (4)
C3	0.0109 (5)	0.0164 (5)	0.0131 (5)	0.0036 (4)	0.0013 (4)	0.0067 (4)
C4	0.0149 (5)	0.0153 (5)	0.0139 (5)	0.0018 (4)	0.0021 (4)	0.0065 (4)
C5	0.0177 (6)	0.0149 (5)	0.0139 (5)	0.0028 (4)	−0.0002 (4)	0.0050 (4)
C6	0.0228 (6)	0.0192 (6)	0.0109 (5)	0.0047 (5)	0.0023 (4)	0.0060 (5)
C7	0.0185 (6)	0.0218 (6)	0.0149 (6)	0.0039 (5)	0.0049 (4)	0.0098 (5)
C8	0.0135 (5)	0.0148 (5)	0.0122 (5)	0.0037 (4)	0.0015 (4)	0.0062 (4)
C9	0.0128 (5)	0.0168 (5)	0.0140 (5)	0.0025 (4)	0.0010 (4)	0.0067 (5)
C10	0.0246 (6)	0.0227 (6)	0.0143 (6)	−0.0033 (5)	−0.0031 (5)	0.0061 (5)
C11	0.0134 (5)	0.0149 (5)	0.0134 (5)	0.0022 (4)	0.0015 (4)	0.0059 (4)
C12	0.0138 (5)	0.0140 (5)	0.0149 (5)	0.0011 (4)	0.0009 (4)	0.0063 (4)
C13	0.0129 (5)	0.0150 (5)	0.0133 (5)	0.0033 (4)	0.0012 (4)	0.0059 (4)
C14	0.0146 (5)	0.0181 (6)	0.0164 (6)	0.0001 (4)	0.0015 (4)	0.0082 (5)
C15	0.0158 (5)	0.0164 (6)	0.0162 (6)	0.0001 (4)	−0.0014 (4)	0.0051 (5)
C16	0.0171 (6)	0.0168 (5)	0.0118 (5)	0.0061 (4)	0.0012 (4)	0.0055 (4)
C17	0.0145 (5)	0.0177 (5)	0.0176 (6)	0.0031 (4)	0.0034 (4)	0.0095 (5)
C18	0.0127 (5)	0.0144 (5)	0.0151 (5)	0.0011 (4)	0.0000 (4)	0.0062 (4)
C19	0.0230 (6)	0.0251 (6)	0.0124 (5)	0.0035 (5)	−0.0021 (5)	0.0022 (5)

### Geometric parameters (Å, º)

**Table:**

O1—C1	1.3438 (15)	C14—C15	1.3944 (17)
O1—C9	1.3794 (12)	C15—C16	1.3939 (19)
O2—C3	1.2345 (17)	C16—C17	1.3985 (14)
O3—C12	1.2315 (15)	C17—C18	1.3782 (17)
O4—C16	1.3631 (16)	O5—H17	0.861
O4—C19	1.4322 (14)	O5—H18	0.871
N1—N2	1.3787 (14)	N2—H6	0.880
N1—C11	1.2827 (13)	C1—H1	0.950
N2—C12	1.3604 (14)	C4—H2	0.950
C1—C2	1.3492 (19)	C6—H3	0.950
C2—C3	1.4586 (14)	C7—H4	0.950
C2—C11	1.4672 (16)	C10—H11A	0.980
C3—C8	1.4732 (16)	C10—H12B	0.980
C4—C5	1.3862 (17)	C10—H13C	0.980
C4—C8	1.4094 (14)	C11—H5	0.950
C5—C6	1.4084 (19)	C14—H7	0.950
C5—C10	1.5074 (15)	C15—H8	0.950
C6—C7	1.3807 (15)	C17—H9	0.950
C7—C9	1.3958 (17)	C18—H10	0.950
C8—C9	1.3916 (18)	C19—H14A	0.980
C12—C13	1.4891 (17)	C19—H15B	0.980
C13—C14	1.3934 (14)	C19—H16C	0.980
C13—C18	1.4014 (19)		
			
O1···C3	2.8584 (17)	C11···H18^iv^	3.0847
O2···C1	3.5695 (17)	C12···H4^i^	3.4658
O2···C4	2.8803 (16)	C12···H11A^xi^	3.3379
O2···C11	2.8936 (14)	C12···H13C^xi^	3.2583
O3···N1	2.7054 (13)	C12···H17^vi^	2.9300
O3···C14	2.9022 (15)	C13···H11A^xi^	3.3981
O3···C18	3.5987 (17)	C13···H14A^viii^	3.0288
N1···C1	2.7788 (17)	C13···H15B^viii^	3.5976
N2···C18	2.8777 (18)	C13···H17^vi^	3.3762
C1···C7	3.5842 (17)	C14···H2^xi^	3.0995
C1···C8	2.7485 (15)	C14···H11A^xi^	3.0798
C2···C9	2.7804 (17)	C14···H14A^viii^	3.4949
C4···C7	2.7985 (19)	C14···H17^vi^	3.2493
C5···C9	2.7814 (15)	C14···H18^vi^	3.3794
C6···C8	2.7899 (17)	C15···H2^xi^	3.1928
C11···C12	3.4855 (17)	C15···H8^xv^	3.4404
C13···C16	2.7930 (18)	C16···H2^iv^	3.4036
C14···C17	2.771 (2)	C17···H1^v^	3.5568
C15···C18	2.7948 (16)	C17···H2^iv^	3.0193
C15···C19	2.8103 (18)	C17···H14A^viii^	3.1958
O1···O1^i^	3.0524 (12)	C18···H14A^viii^	2.8568
O1···C1^i^	3.1131 (15)	C18···H18	3.1229
O1···C3^ii^	3.5514 (17)	C19···H6^viii^	3.4870
O1···C8^ii^	3.5633 (16)	C19···H7^xv^	3.5754
O1···C17^iii^	3.413 (2)	C19···H11A^iv^	3.0741
O2···O5^iv^	2.8274 (16)	C19···H17^viii^	3.5419
O2···C14^v^	3.4847 (18)	C19···H18^viii^	3.2485
O2···C15^v^	3.5467 (17)	H1···O1^i^	2.6612
O2···C17^iv^	3.0969 (15)	H1···O4^iii^	3.4948
O2···C18^iv^	3.2582 (16)	H1···C1^i^	3.4987
O3···O5^vi^	2.8465 (13)	H1···C4^ii^	3.2645
O3···C6^i^	3.4626 (18)	H1···C5^ii^	3.4193
O3···C7^i^	3.2379 (18)	H1···C7^i^	3.4139
O4···C9^v^	3.3715 (18)	H1···C9^i^	3.4026
O5···O2^iv^	2.8274 (16)	H1···C17^v^	3.5568
O5···O3^vii^	2.8465 (13)	H1···H1^i^	3.5510
O5···N2	2.9341 (14)	H1···H2^ii^	3.3061
O5···C11	3.4460 (16)	H1···H4^i^	2.8965
O5···C11^iv^	3.5818 (19)	H1···H9^iii^	2.9731
O5···C18	3.3885 (16)	H1···H9^v^	3.4140
O5···C19^viii^	3.1664 (17)	H1···H12B^ii^	3.4139
N1···C12^v^	3.534 (2)	H2···O4^iv^	3.0040
N1···C13^v^	3.4362 (19)	H2···C1^ii^	3.4871
N1···C18^v^	3.1900 (17)	H2···C14^x^	3.0995
N2···O5	2.9341 (14)	H2···C15^x^	3.1928
N2···C12^v^	3.5061 (19)	H2···C16^iv^	3.4036
C1···O1^i^	3.1131 (15)	H2···C17^iv^	3.0193
C1···C4^ii^	3.297 (2)	H2···H1^ii^	3.3061
C1···C8^ii^	3.4666 (19)	H2···H7^x^	2.7132
C1···C17^v^	3.347 (2)	H2···H8^x^	2.8772
C2···C7^ii^	3.5937 (19)	H2···H9^iv^	2.3881
C2···C8^ii^	3.600 (2)	H2···H16C^v^	3.3063
C2···C9^ii^	3.5376 (19)	H3···O3^i^	2.9428
C2···C17^v^	3.4872 (19)	H3···O5^ii^	2.9615
C2···C18^v^	3.5038 (19)	H3···C11^ii^	3.4417
C3···O1^ii^	3.5514 (17)	H3···H5^ii^	3.1313
C3···C9^ii^	3.422 (2)	H3···H6^ii^	3.5413
C3···C15^v^	3.425 (2)	H3···H13C^xii^	2.8860
C3···C16^v^	3.5874 (18)	H3···H15B^xiv^	3.1317
C4···C1^ii^	3.297 (2)	H3···H17^ii^	2.6475
C5···C11^ii^	3.541 (2)	H4···O3^i^	2.5018
C6···O3^i^	3.4626 (18)	H4···N1^i^	3.1154
C6···C11^ii^	3.300 (2)	H4···C12^i^	3.4658
C7···O3^i^	3.2379 (18)	H4···H1^i^	2.8965
C7···C2^ii^	3.5937 (19)	H4···H5^ii^	3.4550
C8···O1^ii^	3.5633 (16)	H4···H9^iii^	3.5172
C8···C1^ii^	3.4666 (19)	H4···H10^iii^	2.9367
C8···C2^ii^	3.600 (2)	H4···H13C^xii^	3.5497
C9···O4^v^	3.3715 (18)	H4···H17^ii^	3.3353
C9···C2^ii^	3.5376 (19)	H5···O3^v^	3.5370
C9···C3^ii^	3.422 (2)	H5···O5	2.7040
C11···O5	3.4460 (16)	H5···O5^iv^	2.8429
C11···O5^iv^	3.5818 (19)	H5···C6^ii^	3.1539
C11···C5^ii^	3.541 (2)	H5···C7^ii^	3.3413
C11···C6^ii^	3.300 (2)	H5···H3^ii^	3.1313
C11···C13^v^	3.408 (2)	H5···H4^ii^	3.4550
C11···C18^v^	3.4611 (18)	H5···H17	2.8769
C12···N1^v^	3.534 (2)	H5···H17^iv^	2.7727
C12···N2^v^	3.5061 (19)	H5···H18	3.4278
C13···N1^v^	3.4362 (19)	H5···H18^iv^	2.4418
C13···C11^v^	3.408 (2)	H6···O3^v^	3.5738
C14···O2^v^	3.4847 (18)	H6···O5	2.0733
C15···O2^v^	3.5467 (17)	H6···C19^viii^	3.4870
C15···C3^v^	3.425 (2)	H6···H3^ii^	3.5413
C16···C3^v^	3.5874 (18)	H6···H12B^ii^	3.3595
C17···O1^ix^	3.413 (2)	H6···H14A^viii^	2.9852
C17···O2^iv^	3.0969 (15)	H6···H15B^viii^	3.0796
C17···C1^v^	3.347 (2)	H6···H17	2.7998
C17···C2^v^	3.4872 (19)	H6···H18	2.5496
C18···O2^iv^	3.2582 (16)	H7···O2^v^	3.5098
C18···O5	3.3885 (16)	H7···O5^vi^	3.2029
C18···N1^v^	3.1900 (17)	H7···C4^xi^	3.0931
C18···C2^v^	3.5038 (19)	H7···C5^xi^	3.2491
C18···C11^v^	3.4611 (18)	H7···C10^xi^	3.0983
C19···O5^viii^	3.1664 (17)	H7···C19^xv^	3.5754
O1···H4	2.5251	H7···H2^xi^	2.7132
O2···H2	2.6134	H7···H8^xv^	3.3791
O2···H5	2.7069	H7···H11A^xi^	2.7136
O3···H6	3.0551	H7···H13C^xi^	2.9516
O3···H7	2.6534	H7···H14A^xv^	3.5658
O4···H8	2.6732	H7···H16C^xv^	2.7801
O4···H9	2.4801	H7···H17^vi^	2.7717
N1···H1	2.4498	H7···H18^vi^	3.0174
N2···H5	2.3906	H8···O2^xi^	3.1894
N2···H10	2.6248	H8···C4^xi^	3.5614
C1···H5	3.2679	H8···C15^xv^	3.4404
C3···H1	3.2802	H8···H2^xi^	2.8772
C3···H2	2.6884	H8···H7^xv^	3.3791
C3···H5	2.7576	H8···H8^xv^	2.7580
C4···H3	3.2557	H9···O1^ix^	2.6230
C4···H11A	2.5696	H9···O1^v^	3.4596
C4···H12B	3.1436	H9···O2^iv^	2.6002
C4···H13C	3.1436	H9···C1^ix^	3.1313
C5···H4	3.2972	H9···C1^v^	3.3502
C6···H2	3.2605	H9···C3^iv^	3.2617
C6···H11A	3.3234	H9···C4^iv^	3.0509
C6···H12B	2.7605	H9···C8^iv^	3.4940
C6···H13C	2.7782	H9···C9^ix^	3.5961
C8···H4	3.2968	H9···H1^ix^	2.9731
C9···H1	3.1875	H9···H1^v^	3.4140
C9···H2	3.2584	H9···H2^iv^	2.3881
C9···H3	3.2399	H9···H4^ix^	3.5172
C10···H2	2.6780	H10···O2^iv^	2.8992
C10···H3	2.6698	H10···O3^v^	3.2467
C11···H1	2.5457	H10···O5	2.8202
C11···H6	2.4080	H10···N1^v^	3.0018
C12···H7	2.6281	H10···H4^ix^	2.9367
C12···H10	2.6974	H10···H14A^viii^	3.0995
C13···H6	2.4962	H10···H18	2.5593
C13···H8	3.2867	H11A···O3^x^	3.5087
C13···H9	3.2692	H11A···O4^iv^	2.7373
C14···H10	3.2651	H11A···C12^x^	3.3379
C15···H9	3.2762	H11A···C13^x^	3.3981
C15···H14A	2.6970	H11A···C14^x^	3.0798
C15···H16C	2.7835	H11A···C19^iv^	3.0741
C16···H7	3.2575	H11A···H7^x^	2.7136
C16···H10	3.2648	H11A···H12B^xiii^	3.0810
C16···H14A	2.5828	H11A···H14A^iv^	3.4356
C16···H15B	3.1948	H11A···H15B^iv^	2.5934
C16···H16C	2.6505	H12B···N1^ii^	3.0093
C17···H8	3.2812	H12B···N2^ii^	3.1500
C18···H6	2.5794	H12B···C10^xiii^	3.1944
C18···H7	3.2633	H12B···C11^ii^	3.4557
C19···H8	2.5204	H12B···H1^ii^	3.4139
H1···H5	3.4530	H12B···H6^ii^	3.3595
H2···H11A	2.3603	H12B···H11A^xiii^	3.0810
H2···H12B	3.3527	H12B···H12B^xiii^	2.8819
H2···H13C	3.3240	H12B···H13C^xiii^	3.0808
H3···H4	2.3310	H12B···H15B^xiv^	2.7976
H3···H12B	2.7103	H12B···H15B^iv^	3.4853
H3···H13C	2.7079	H13C···O3^x^	2.8693
H5···H6	2.1974	H13C···C6^xii^	3.4168
H6···H10	2.1755	H13C···C12^x^	3.2583
H7···H8	2.3430	H13C···H3^xii^	2.8860
H8···H14A	2.2623	H13C···H4^xii^	3.5497
H8···H15B	3.4901	H13C···H7^x^	2.9516
H8···H16C	2.3453	H13C···H12B^xiii^	3.0808
H9···H10	2.3234	H13C···H15B^xiv^	3.0074
O1···H1^i^	2.6612	H14A···O2^xi^	3.2341
O1···H9^iii^	2.6230	H14A···O5^viii^	2.6683
O1···H9^v^	3.4596	H14A···N2^viii^	3.5424
O2···H7^v^	3.5098	H14A···C13^viii^	3.0288
O2···H8^x^	3.1894	H14A···C14^viii^	3.4949
O2···H9^iv^	2.6002	H14A···C17^viii^	3.1958
O2···H10^iv^	2.8992	H14A···C18^viii^	2.8568
O2···H14A^x^	3.2341	H14A···H6^viii^	2.9852
O2···H17^iv^	3.1820	H14A···H7^xv^	3.5658
O2···H18^iv^	1.9585	H14A···H10^viii^	3.0995
O3···H3^i^	2.9428	H14A···H11A^iv^	3.4356
O3···H4^i^	2.5018	H14A···H17^viii^	3.2070
O3···H5^v^	3.5370	H14A···H18^viii^	2.5780
O3···H6^v^	3.5738	H15B···O5^viii^	3.0892
O3···H10^v^	3.2467	H15B···N2^viii^	3.4220
O3···H11A^xi^	3.5087	H15B···C10^xvi^	3.3329
O3···H13C^xi^	2.8693	H15B···C10^iv^	3.4241
O3···H17^vi^	1.9860	H15B···C13^viii^	3.5976
O3···H18^vi^	3.1614	H15B···H3^xvi^	3.1317
O4···H1^ix^	3.4948	H15B···H6^viii^	3.0796
O4···H2^iv^	3.0040	H15B···H11A^iv^	2.5934
O4···H11A^iv^	2.7373	H15B···H12B^xvi^	2.7976
O5···H3^ii^	2.9615	H15B···H12B^iv^	3.4853
O5···H5	2.7040	H15B···H13C^xvi^	3.0074
O5···H5^iv^	2.8429	H15B···H17^viii^	3.4492
O5···H6	2.0733	H15B···H18^viii^	3.4212
O5···H7^vii^	3.2029	H16C···O5^viii^	3.2344
O5···H10	2.8202	H16C···C4^v^	2.9199
O5···H14A^viii^	2.6683	H16C···C5^v^	3.0378
O5···H15B^viii^	3.0892	H16C···C6^v^	3.2428
O5···H16C^viii^	3.2344	H16C···C7^v^	3.3992
N1···H4^i^	3.1154	H16C···C8^v^	3.0929
N1···H10^v^	3.0018	H16C···C9^v^	3.3280
N1···H12B^ii^	3.0093	H16C···H2^v^	3.3063
N2···H12B^ii^	3.1500	H16C···H7^xv^	2.7801
N2···H14A^viii^	3.5424	H16C···H17^viii^	3.3865
N2···H15B^viii^	3.4220	H16C···H18^viii^	3.2768
N2···H18	3.4279	H17···O2^iv^	3.1820
C1···H1^i^	3.4987	H17···O3^vii^	1.9860
C1···H2^ii^	3.4871	H17···C6^ii^	3.3703
C1···H9^iii^	3.1313	H17···C11^iv^	3.4096
C1···H9^v^	3.3502	H17···C12^vii^	2.9300
C2···H18^iv^	3.4662	H17···C13^vii^	3.3762
C3···H9^iv^	3.2617	H17···C14^vii^	3.2493
C3···H18^iv^	2.9714	H17···C19^viii^	3.5419
C4···H1^ii^	3.2645	H17···H3^ii^	2.6475
C4···H7^x^	3.0931	H17···H4^ii^	3.3353
C4···H8^x^	3.5614	H17···H5	2.8769
C4···H9^iv^	3.0509	H17···H5^iv^	2.7727
C4···H16C^v^	2.9199	H17···H6	2.7998
C5···H1^ii^	3.4193	H17···H7^vii^	2.7717
C5···H7^x^	3.2491	H17···H14A^viii^	3.2070
C5···H16C^v^	3.0378	H17···H15B^viii^	3.4492
C6···H5^ii^	3.1539	H17···H16C^viii^	3.3865
C6···H13C^xii^	3.4168	H18···O2^iv^	1.9585
C6···H16C^v^	3.2428	H18···O3^vii^	3.1614
C6···H17^ii^	3.3703	H18···N2	3.4279
C7···H1^i^	3.4139	H18···C2^iv^	3.4662
C7···H5^ii^	3.3413	H18···C3^iv^	2.9714
C7···H16C^v^	3.3992	H18···C11^iv^	3.0847
C8···H9^iv^	3.4940	H18···C14^vii^	3.3794
C8···H16C^v^	3.0929	H18···C18	3.1229
C9···H1^i^	3.4026	H18···C19^viii^	3.2485
C9···H9^iii^	3.5961	H18···H5	3.4278
C9···H16C^v^	3.3280	H18···H5^iv^	2.4418
C10···H7^x^	3.0983	H18···H6	2.5496
C10···H12B^xiii^	3.1944	H18···H7^vii^	3.0174
C10···H15B^xiv^	3.3329	H18···H10	2.5593
C10···H15B^iv^	3.4241	H18···H14A^viii^	2.5780
C11···H3^ii^	3.4417	H18···H15B^viii^	3.4212
C11···H12B^ii^	3.4557	H18···H16C^viii^	3.2768
C11···H17^iv^	3.4096		
			
C1—O1—C9	118.30 (11)	C16—C17—C18	120.28 (13)
C16—O4—C19	116.81 (11)	C13—C18—C17	120.42 (9)
N2—N1—C11	115.03 (10)	H17—O5—H18	104.049
N1—N2—C12	119.09 (10)	N1—N2—H6	120.456
O1—C1—C2	125.30 (9)	C12—N2—H6	120.455
C1—C2—C3	119.92 (10)	O1—C1—H1	117.348
C1—C2—C11	119.51 (9)	C2—C1—H1	117.351
C3—C2—C11	120.57 (11)	C5—C4—H2	119.347
O2—C3—C2	123.39 (10)	C8—C4—H2	119.353
O2—C3—C8	122.09 (9)	C5—C6—H3	119.062
C2—C3—C8	114.51 (11)	C7—C6—H3	119.061
C5—C4—C8	121.30 (12)	C6—C7—H4	120.966
C4—C5—C6	118.44 (10)	C9—C7—H4	120.967
C4—C5—C10	121.41 (12)	C5—C10—H11A	109.468
C6—C5—C10	120.13 (11)	C5—C10—H12B	109.473
C5—C6—C7	121.88 (11)	C5—C10—H13C	109.474
C6—C7—C9	118.07 (12)	H11A—C10—H12B	109.466
C3—C8—C4	121.97 (11)	H11A—C10—H13C	109.475
C3—C8—C9	120.11 (9)	H12B—C10—H13C	109.472
C4—C8—C9	117.92 (11)	N1—C11—H5	120.708
O1—C9—C7	115.96 (11)	C2—C11—H5	120.713
O1—C9—C8	121.73 (10)	C13—C14—H7	119.332
C7—C9—C8	122.31 (9)	C15—C14—H7	119.329
N1—C11—C2	118.58 (11)	C14—C15—H8	120.561
O3—C12—N2	122.85 (11)	C16—C15—H8	120.551
O3—C12—C13	122.70 (10)	C16—C17—H9	119.859
N2—C12—C13	114.45 (10)	C18—C17—H9	119.864
C12—C13—C14	119.21 (12)	C13—C18—H10	119.788
C12—C13—C18	121.95 (9)	C17—C18—H10	119.793
C14—C13—C18	118.82 (11)	O4—C19—H14A	109.469
C13—C14—C15	121.34 (13)	O4—C19—H15B	109.476
C14—C15—C16	118.89 (10)	O4—C19—H16C	109.470
O4—C16—C15	124.87 (9)	H14A—C19—H15B	109.472
O4—C16—C17	114.91 (12)	H14A—C19—H16C	109.471
C15—C16—C17	120.21 (11)	H15B—C19—H16C	109.469
			
C1—O1—C9—C7	−178.27 (10)	C6—C5—C10—H11A	178.6
C1—O1—C9—C8	1.07 (17)	C6—C5—C10—H12B	58.6
C9—O1—C1—C2	−2.91 (18)	C6—C5—C10—H13C	−61.4
C9—O1—C1—H1	177.1	C10—C5—C6—C7	−176.19 (11)
C16—O4—C19—H14A	−54.8	C10—C5—C6—H3	3.8
C16—O4—C19—H15B	−174.8	C5—C6—C7—C9	0.2 (2)
C16—O4—C19—H16C	65.2	C5—C6—C7—H4	−179.8
C19—O4—C16—C15	−3.73 (17)	H3—C6—C7—C9	−179.8
C19—O4—C16—C17	177.18 (10)	H3—C6—C7—H4	0.2
N2—N1—C11—C2	−178.93 (10)	C6—C7—C9—O1	176.77 (11)
N2—N1—C11—H5	1.1	C6—C7—C9—C8	−2.58 (19)
C11—N1—N2—C12	−169.22 (11)	H4—C7—C9—O1	−3.2
C11—N1—N2—H6	10.8	H4—C7—C9—C8	177.4
N1—N2—C12—O3	−4.41 (19)	C3—C8—C9—O1	2.10 (18)
N1—N2—C12—C13	175.96 (10)	C3—C8—C9—C7	−178.59 (10)
H6—N2—C12—O3	175.6	C4—C8—C9—O1	−177.05 (10)
H6—N2—C12—C13	−4.0	C4—C8—C9—C7	2.26 (18)
O1—C1—C2—C3	1.3 (2)	O3—C12—C13—C14	−30.86 (18)
O1—C1—C2—C11	−177.80 (11)	O3—C12—C13—C18	147.39 (12)
H1—C1—C2—C3	−178.7	N2—C12—C13—C14	148.77 (11)
H1—C1—C2—C11	2.2	N2—C12—C13—C18	−32.97 (17)
C1—C2—C3—O2	−178.28 (12)	C12—C13—C14—C15	−179.76 (11)
C1—C2—C3—C8	1.82 (17)	C12—C13—C14—H7	0.2
C1—C2—C11—N1	21.42 (18)	C12—C13—C18—C17	−178.28 (10)
C1—C2—C11—H5	−158.6	C12—C13—C18—H10	1.7
C3—C2—C11—N1	−157.72 (11)	C14—C13—C18—C17	−0.02 (18)
C3—C2—C11—H5	22.3	C14—C13—C18—H10	180.0
C11—C2—C3—O2	0.86 (18)	C18—C13—C14—C15	1.93 (19)
C11—C2—C3—C8	−179.04 (10)	C18—C13—C14—H7	−178.1
O2—C3—C8—C4	−4.21 (19)	C13—C14—C15—C16	−2.29 (19)
O2—C3—C8—C9	176.67 (11)	C13—C14—C15—H8	177.7
C2—C3—C8—C4	175.68 (10)	H7—C14—C15—C16	177.7
C2—C3—C8—C9	−3.43 (16)	H7—C14—C15—H8	−2.3
C5—C4—C8—C3	−178.65 (11)	C14—C15—C16—O4	−178.30 (11)
C5—C4—C8—C9	0.48 (18)	C14—C15—C16—C17	0.75 (19)
C8—C4—C5—C6	−2.74 (18)	H8—C15—C16—O4	1.7
C8—C4—C5—C10	175.85 (10)	H8—C15—C16—C17	−179.3
H2—C4—C5—C6	177.3	O4—C16—C17—C18	−179.74 (10)
H2—C4—C5—C10	−4.1	O4—C16—C17—H9	0.3
H2—C4—C8—C3	1.3	C15—C16—C17—C18	1.12 (19)
H2—C4—C8—C9	−179.5	C15—C16—C17—H9	−178.9
C4—C5—C6—C7	2.42 (19)	C16—C17—C18—C13	−1.49 (19)
C4—C5—C6—H3	−177.6	C16—C17—C18—H10	178.5
C4—C5—C10—H11A	−0.0	H9—C17—C18—C13	178.5
C4—C5—C10—H12B	−120.0	H9—C17—C18—H10	−1.5
C4—C5—C10—H13C	120.0		

Symmetry codes: (i) −*x*, −*y*+1, −*z*+1; (ii) −*x*+1, −*y*+1, −*z*+1; (iii) *x*, *y*, *z*+1; (iv) −*x*+1, −*y*+1, −*z*; (v) −*x*, −*y*+1, −*z*; (vi) *x*−1, *y*, *z*; (vii) *x*+1, *y*, *z*; (viii) −*x*, −*y*, −*z*−1; (ix) *x*, *y*, *z*−1; (x) *x*+1, *y*+1, *z*+1; (xi) *x*−1, *y*−1, *z*−1; (xii) −*x*+1, −*y*+2, −*z*+2; (xiii) −*x*+2, −*y*+2, −*z*+2; (xiv) *x*+1, *y*+1, *z*+2; (xv) −*x*−1, −*y*, −*z*−1; (xvi) *x*−1, *y*−1, *z*−2.

### Hydrogen-bond geometry (Å, º)

**Table:**

*D*—H···*A*	*D*—H	H···*A*	*D*···*A*	*D*—H···*A*
O5—H17···O3^vii^	0.86	1.99	2.8465 (13)	178
O5—H18···O2^iv^	0.87	1.96	2.8274 (16)	176
N2—H6···O5	0.88	2.07	2.9341 (14)	166

Symmetry codes: (iv) −*x*+1, −*y*+1, −*z*; (vii) *x*+1, *y*, *z*.
